# MnO/ZnO:Zn Thin-Film Frequency Adaptive Heterostructure for Future Sustainable Memristive Systems

**DOI:** 10.3390/nano14080659

**Published:** 2024-04-10

**Authors:** Karen A. Neri-Espinoza, José A. Andraca-Adame, Miguel A. Domínguez-Crespo, Francisco Gutiérrez-Galicia, Roberto Baca-Arroyo, Héctor J. Dorantes-Rosales, Ramón Peña-Sierra

**Affiliations:** 1 Department of Nanomaterials, Unidad Profesional Interdisciplinaria de Ingeniería Campus Hidalgo (UPIIH), Instituto Politécnico Nacional (IPN), Hidalgo 42162, Mexico; mdominguezc@ipn.mx (M.A.D.-C.); fgutierrezga@ipn.mx (F.G.-G.); 2 Department of Electronics, Escuela Superior de Ingeniería Mecánica y Eléctrica (ESIME), Instituto Politécnico Nacional (IPN), Mexico City 07738, Mexico; rbaca02006@yahoo.com.mx; 3 Department of Metallurgical and Materials Engineering, Escuela Superior de Ingeniería Química e Industrias Extractivas (ESIQIE), Instituto Politécnico Nacional (IPN), Mexico City 07738, Mexico; hectordorantes@yahoo.com; 4 Department of Electrical Engineering, Sección de Electrónica de Estado Sólido (SEES), Center for Research and Advanced Studies of the National Polytechnic Institute (CINVESTAV-IPN), Mexico City 07360, Mexico; rpsierra@cinvestav.mx

**Keywords:** MnO, ZnO:Zn, memristor, I–V curves, adaptive electronics, sustainable electronics

## Abstract

In recent years, advances in materials engineering based on adaptive electronics have found a new paradigm to optimize drawbacks in signal processing. A two-layer MnO/ZnO:Zn heterostructure envisioned for frequency adaptive electronic signal processing is synthesized by sputtering, where the use of internal states allows reconfigurability to obtain new operating modes at different frequency input signals. X-ray diffraction (XRD) analysis is performed on each layer, revealing a cubic structure for MnO and a hexagonal structure for ZnO:Zn with preferential growth in [111] and [002] directions, respectively. Scanning electron microscope (SEM) micrographs show that the surface of both materials is homogeneous and smooth. The thickness for each layer is determined to be approximately 106.3 nm for MnO, 119.3 nm for ZnO:Zn and 224.1 nm for the MnO/ZnO:Zn structure. An electrical characterisation with an oscilloscope and signal generator was carried out to obtain the time-response signals and current-voltage (I–V) curves, where no degradation is detected when changing frequencies within the range of 100 Hz to 1 MHz. An equivalent circuit is proposed to explain the effects in the interface. Measurements of switching speeds from high resistance state (HRS) to low resistance state (LRS) at approximately 17 ns, highlight the device’s rapid adaptability, and an estimated switching ratio of approximately 2 × 10^4^ indicates its efficiency as a memristive component. Finally, the MnO/ZnO:Zn heterojunction delivers states that are stable, repeatable, and reproducible, demonstrating how the interaction of the materials can be utilised in adaptive device applications by applying frequencies and internal states to create new and innovative design schematics, thus reducing the number of components/connections in a system for future sustainable electronics.

## 1. Introduction

Different approaches of signal processing are being developed largely driven by Moore’s law [1,2]. One of these approaches is through adaptive electronics. An adaptive device can be defined as an electronic structure designed to incorporate internal mechanisms (states) capable of reconfiguration, enabling new operating modes. The structure’s reaction to an external stimulus (such as a change in frequency) will self-adjust the parameters of those properties to carry out certain electronic operations [3,4,5].Therefore, frequency-adaptive electronic signal processing devices can be considered as a feasible solution for the future of high-speed and low-power electronic devices in which dynamic self-adjusting parameters can be reconfigured by manipulating its properties. Examples of adaptive behaviour can include the use of polarization (spintronics), oxide attributes (multiferroics, piezoelectricity, strain), modifications in dimensions (thickness and area), and nonlinear conduction (memristors) [6,7,8,9,10,11]. Memristive behaviour (memristor is a combination of the words “memory” and “resistor”) [12] are typically found on metal oxides where the switching of different states can be manipulated with electronic transport mechanisms such as migration of oxygen vacancies, conduction through filament paths, and capacitive-inductive effects [13,14,15,16,17]. A main characteristic of an ideal memristor is a zero-crossing pinched current-voltage (I–V) hysteresis curve (I = 0 and V = 0). Several previous works have found that the interaction of heterojunctions of transition metal oxides (TMOs) can create non-zero-crossing points in the I–V curve [10,16,18,19]. This phenomenon can occur for various reasons [10,16] such as inductive and capacitive effects, indicating a need for expanding the memristive theory to assign more coupled effects and new applications.

A number of TMO structures involving ZnO and MnO_x_ (such as MnO_x_/HfO_x_ [20], Pt/MnO_x_/Pt [21], Pt/ZnO/Pt [22]) have reported resistive switching characteristics.

Table 1 presents a brief comparison of memristive devices of different materials, showing key attributes such as thickness, conduction mechanisms, I–V curve types, threshold voltages, switching ratios, and switching speeds. The threshold voltage and switching ratio serve as critical indicators of the energy requirements and data distinction capabilities of these devices, while the switching speed focuses on the performance in high-speed applications. Commercial memristors typically have switching speeds in the range of 50 ns–100 µs [23]. A more profound study of switching mechanisms is needed to fully understand how speed affects application performance. As can be seen from Table 1 and other comparison tables reported [11,24,25,26,27], most structures consist of three or more layers/interfaces. This reflects greater complexity for the synthesis process and likewise, the transport mechanisms become more intricate to study. The proposed bilayer structure reduces the memristive response to one interface and two materials with their oxides. Due to the simplicity of the structure, it is possible to propose an equivalent circuit to explore how the interface could be translated to already known electronic components, with the purpose of understanding the internal switching adaptive mechanisms through frequency variation. In this work, switching is proposed to occur with the variation of frequency in order to control the states.

These behaviours and parameters are of great interest to the development of neuromorphic materials as well as new applications for sustainable electronics [28,29,30,31], as different transport mechanisms in the interface can help reduce the number of elements in a structure, making it efficient, reconfigurable, responsive, and low power where components can be passive (resistors, inductors, capacitors) or active (transistors, diodes).

In this paper, a simple MnO/ZnO:Zn bilayer thin-film heterostructure is synthesized by the sputtering technique to improve electronic signal processing as a frequency adaptive memristive system. The ZnO has a direct wide bandgap of approximately 3.3 eV, and its most common potential applications are for laser diodes, light-emitting diodes (LEDs) [32], and transparent thin-film transistors (TTFT). Furthermore, Zn-doped ZnO (ZnO:Zn) can be used as a thin-film to design structures to drive the electrical responses using transport and interface phenomena [6,13,15,26]. The Mn and its oxides continue to be an inorganic material of technological importance for environmental remediation, electrochemical capacitors [33,34,35] as well as metal oxide-based RRAM devices due to the defects, vacancies and oxidation propensity of MnO_x_ [36,37]. Many of the reported memristive systems are thin-films prepared by different methods such as pulsed laser deposition (PLD), chemical vapour deposition (CVD), and electrochemical and magnetron sputtering deposition. The sputtering offers repeatable, reproducible, scalable, uniform, and high-quality films. These properties are needed to form stable heterostructures/heterojunctions, and this work considers previously determined experimental conditions [38,39].

**Table 1:** Comparison of different materials memristive devices and their reported characteristics.

Materials	Thickness of Structure	Conduction Mechanism	I–V Curve Type	Threshold Voltage (V)	Switching Ratio	Switching Speed (ns)	Ref.
Pt/Ta_2_O_5−x_/TaO_2−x_/Pt	~110 nm	Oxygen vacancies	Zero crossing	1	>10	10	[40]
Ag/MnO_x_/Pt	~282 nm	Ag filament or by oxygen vacancies	Zero crossing	0.95	~3.2 × 10^3^	-	[20]
Ag/HfO_y_/Pt	~282 nm	Bipolar resistive switching	Zero crossing	0.45	8.21 × 10^4^	-	[20]
Ag/MnO_x_/HfO_y_/Pt	~282 nm	Bipolar resistive switching	Zero crossing	0.65	6.91 × 10^5^	-	[20]
Pt/MnO_x_/Pt	~250 nm	Ohmic conduction	Non-zero crossing	12	>10^3^	100	[21]
Pt/MnOx/Al	~250 nm	Ohmic conduction	Non-zero crossing	0.3	>10^3^	100	[21]
Ag–ZnO/ZnSnO_3_–Ag	ZnO NW’s (100 nm diameter, 0.5 mm length)	Bipolar resistive switching	Non-zero crossing	1.7	5.8 × 10^2^	-	[41]
Pt/ZnO/Pt	~220 µm 100 nm for ZnO	Bipolar resistive switching Filament	Zero crossing	3.3	10^3^–10^4^	-	[22]
MnO/ZnO:Zn	~200 nm	Bipolar resistive switching Filament	Non-zero crossing (f > 100 kHz)	0.44	~2.11 × 10^4^	~17	This work

For the MnO/ZnO:Zn heterostructure of this project, morphological and structural characterisations were carried out by X-ray diffraction (XRD), scanning electron microscopy (SEM), and energy dispersive X-ray spectroscopy (EDS), and were discussed in terms of the electrical performance. The electrical response of the films is studied in the frequency range of 100 Hz to 1 MHz with a digital oscilloscope and function generator. The adaptability of the system through different frequencies and the combination of the proposed metal oxides were analysed in this manuscript to determine potential applications as a frequency-adaptive structure for future sustainable memristive electronic systems.

## 2. Materials and Methods

The synthesis is crucial to obtain the desired characteristics of the memristive system. For this reason, a detailed explanation of the substrate cleaning procedure, as well as the deposition by sputtering, is provided below.

### 2.1. Substrate Cleaning Procedure

**Figure 1:**
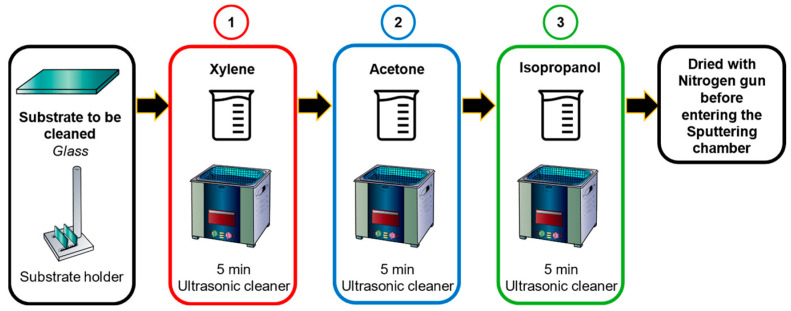
Substrate cleaning process before sputtering deposition.

To remove contaminants on the surface that can lead to defects in the thin-film, affecting its properties, the cleaning of the glass substrate before the deposition is a critical step to ensure the quality and performance of the heterostructure. The cleaning process (as seen in Figure 1) typically involves sonication of the substrate in each reagent, followed by drying it with nitrogen.

This process ensures that the glass substrate is free from organic and inorganic residues, fingerprints, dust particles, and other contaminants that could affect the adhesion and uniformity of the thin-films.

### 2.2. Thin-Film Deposition by Sputtering

The Mn and ZnO:Zn samples were synthesized using a physical vapor deposition (PVD) sputtering system from Kurt J. Lesker (Jefferson Hills, PA, USA) with a configuration of two sputter gun sources, DC and RF. The vacuum chamber is evacuated by a mechanical pump for 2 h and later by a turbomolecular pump for 5 min to achieve a pressure of 2.5 mTorr. After that, ultra-high purity (99.999%) Argon (Ar) gas was introduced in the chamber. The targets used are 2″ in diameter and 0.256″ in thickness; Mn of 99.9%, ZnO of 99.99% and Zn of 99.999% purity. The deposition process was carried out at room temperature and in different stages to guarantee the desires thickness (100 nm) of each film.

In the first stage, the Mn target was used with DC Sputtering source at 30 W and 5 SCCM (Standard Cubic Centimeters per Minute) of Ar with a working pressure of 3.5 mTorr for 90 min.

The next step considers a ZnO:Zn film co-deposition using a RF/DC configuration. The applied power was 125 W for ZnO (RF source) and 5 W for Zn (DC source) with a working pressure of 5 mTorr, Ar flow of 10 SCCM, and a deposition time of 25 min. These conditions have been optimised [39] and offer interesting electrical responses for adaptive devices. Both stages were carried out several times to ensure repeatability and reproducibility. Each set of samples were studied separately and are labelled as MnGl and ZnGl (Gl meaning “on glass”). Table 2 presents the summarized deposition conditions for each layer.

**Figure 2:**
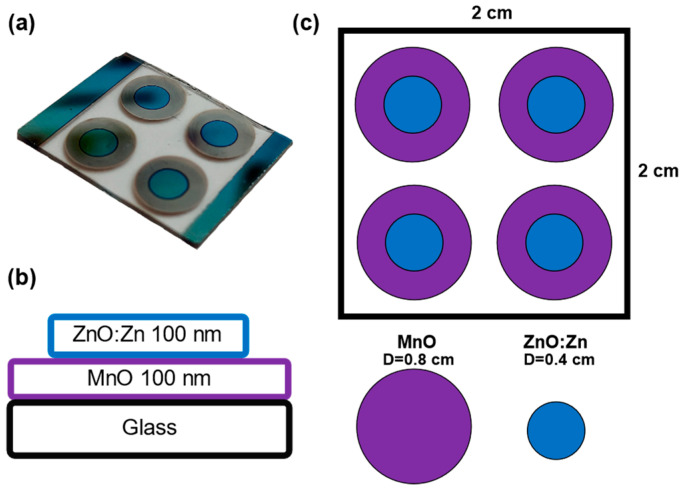
(**a**) Photograph of complete MnO/ZnO heterostructure, (**b**) cross-sectional diagram of structure, indicating thickness of as-prepared films, (**c**) area of deposition showing stainless-steel mask to define each material.

In the final stage, to obtain the bilayer heterostructure, a conventional deposition with a stainless-steel mask was employed. In this process, a grid of circular geometries was used to define the areas of each oxide layer during the deposition (Figure 2).

### 2.3. Characterisations

A profilometer (KLA-TENCOR) was used to corroborate the average thickness of the films.

XRD was performed to evaluate the structural characteristics of the samples synthesized by sputtering. X-ray diffraction patterns (XRDP) were obtained with a PANalytical X’Pert Pro diffractometer (radiation CuKα, λ = 0.15418 nm) in the range of 30–60° with a step size of 0.04° in 2 Theta-Omega (powder) configuration.

The incident optics used were with 1/8 divergent slit, mirror (parallel beam), and 10 mm of mask. A Pixcell ultrafast detector was used with 256 channels to obtain the pattern. The voltage at 45 kV and current at 40 mA were used for the X-ray tube power.

An estimation of the crystallite size for each sample was caried out using the Debye–Scherrer equation (Equation (1)):

(1) $$D=\frac{k\lambda }{\beta cos\theta }$$

D indicates the crystallite size in nm, λ is 0.15418 nm from the CuK_α_ probe of the diffractometer and β is the full-width at half maximum (FWHM) of the peak from XRDP with instrumental correction due to the measurements. HighScore Plus software (version 3.0e) from PANalytical (Malvern, UK) was used to calculated *D*.

The strain (*ε*) of the deposited film was calculated by (Equation (2)):

(2) $$\varepsilon =\frac{d-d_{0}}{d_{0}}\times 100$$

where *d*_0_ is the theoretical interplanar distance for each peak, and *d* is the interplanar distance measured from XRDP.

Scanning electron microscopy (SEM) micrographs of the surface and cross-sections of the samples were obtained with a JEOL JSM-6701F (Tokyo, Japan). An energy dispersive X-ray spectroscopy (EDS) attached to the SEM was used to obtain a semi-quantitative analysis of the elements present in each layer.

For the electrical response of the as-prepared heterostructures, I–V (Current-Voltage) curves were acquired and analysed at different frequencies with a Keysight EDUX1002G oscilloscope (Santa Rosa, CA, USA). A function generator integrated into the oscilloscope was employed to produce the sinusoidal signal to emulate transient polarization at a frequency range of 100 Hz to 1 MHz with voltage from −4 V to 4 V corresponding to the low-level injection.

**Figure 3:**
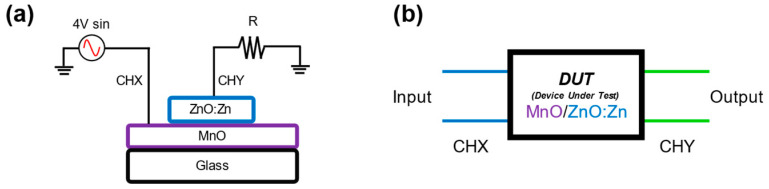
(**a**) Electrical diagram proposed to evaluate MnO/ZnO:Zn heterostructure. CHX and CHY are channels of oscilloscope. (**b**) Heterostructure is a DUT where, with analysis of signals obtained with oscilloscope, adaptive behaviour dependant of frequency can be related to an analogy of a circuit.

Figure 3a shows the electrical diagram used to measure the MnO/ZnO:Zn heterostructure. A sinusoidal signal was connected in series with a 1 kΩ load resistor designated as *R*. The voltage signal across the heterostructure was measured directly as CHX, and the equivalent current was monitored by determining the voltage across the load resistor as CHY. The MnO/ZnO:Zn structure acts as a device under test (DUT) in which we know the input and output, and the inside can be modelled with an equivalent circuit in Figure 3b.

## 3. Results and Discussion

### 3.1. Average Thickness of the Films

The PVD sputtering technique allows for fine control of the thickness in the synthesis process. With the purpose of corroborating the measurements made by the equipment, a profilometer is used in each set of samples using a step made with Kapton^®^ tape before each deposition.

The thickness average was found to be 107.1 ± 9.8 nm for Mn and 116.9 ± 8.4 nm for ZnO:Zn under the sputtering conditions described above. These results are consistent with the information that the sputtering thickness monitor (Inficon SQM-160, Bad Ragaz, Switzerland) exhibits at the end of the synthesis.

### 3.2. XRD

**Figure 4:**
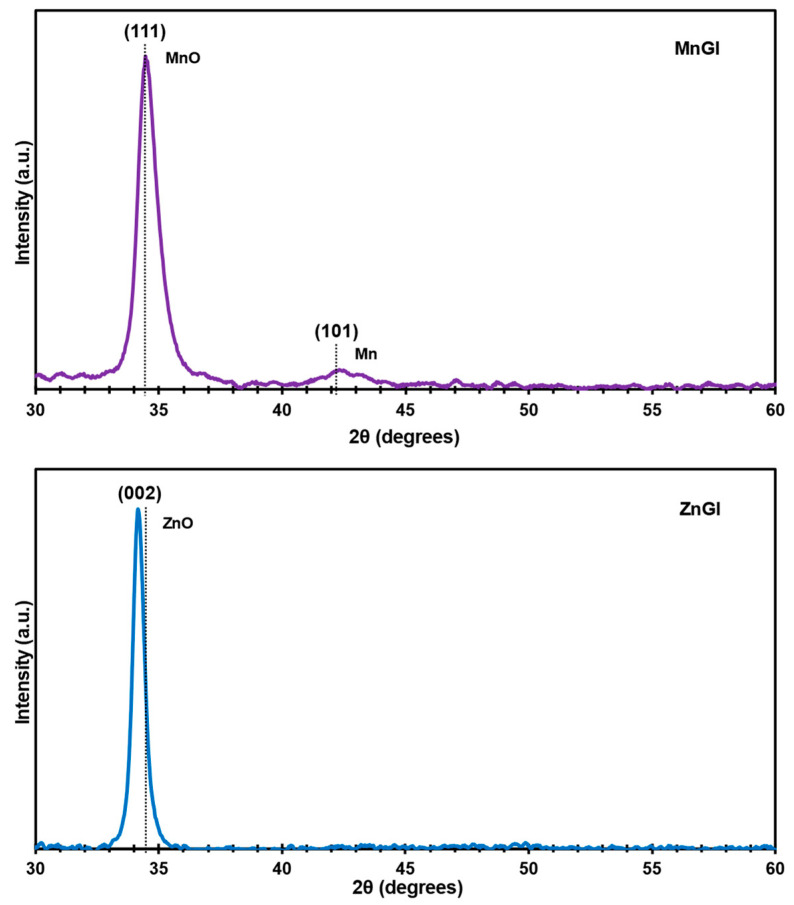
Diffraction patterns for MnGl and ZnGl.

The XRDP of the MnGl and ZnGl films are shown in Figure 4. The MnGl diffraction pattern shows two peaks: the peak at 34.448° corresponds to MnO cubic (111) phase according to the ICDD #98-065-7311 crystallographic chart. The second peak was observed at 42.378°, which is related to the cubic (101) Mn phase (ICDD #00-017-0910).

From this characterisation, two main statements can be established: (1) MnO films grow with a preferential orientation of the crystals at (111); and (2) it is possible to observe a minimal quantity of metallic Mn indicating that, during the sputtering process, the deposited Mn was not fully oxidised and implies the presence of Mn as conglomerates, and it can be described as a MnO:Mn film at the defined synthesis conditions.

The XRDP for the ZnGl film reveals only one peak at 34.133° with a high preferential orientation, corresponding to the plane (002) of ZnO, according with the ICDD #00-036-1451 chart. Structural parameters were calculated using Equations (1) and (2). The results are presented in Table 3.

In a sputtering and co-sputtering process, the deposition of the film is affected by parameters such as pressure, power, and gas flux, which can provoke stress and defects, altering the stability of the lattice, more so, in the case of the ZnO:Zn. To assess the stress in the as-obtained films, Equation (2) was used. The strain (*ε*) results indicate that there are tensile stresses in the perpendicular direction to the (002) plane of ZnO and compression in the parallel direction to the plane of the samples. This deformation is associated to interstitial Zn (Zn_i_), in which the atom of Zn has a radius of 137 pm and is introduced to the ZnO lattice in the co-sputtering process [42,43,44]. Zn_i_ generates an increase in the interplanar distance, which produces defects, stress and strain in the lattice. This inclusion can be linked to an increase in electrical conductivity as the film can be electrically measured (Appendix A). Pure ZnO films display dielectric behaviour whereas the ZnO:Zn system can present an important resistivity decrease [26,45,46,47].

The MnO films presents almost no strain and is conforming to the glass substrate.

Conditions for bilayer thin-films growth of semiconductors (in this case ZnO:Zn on top of MnO) requires that both materials have a close interplanar distance as to avoid generating interfacial defects. For this reason, the parameter Δ*d*/*d* between top and bottom layers were calculated as follows [48]:

(3) $$\frac{\Delta d}{d}=\frac{top\;material-bottom\;material}{bottom\;material}\times 100$$

In this equation, *d* is the interplanar distance from the diffraction patterns for MnO (bottom) and ZnO:Zn (top) respectively. The mismatch is presented on Table 4.

Table 4 displays the differences involving the semiconductor films deposited by sputtering. The Δ*d*/*d*_MnO_ percentage of the synthesized heterostructure is 0.846%. The relation Δ*d*/*d* must be less than 1% to assure a good coupling between the lattices of the materials and to reduce the probability of defects in the interface [48]. The proposed MnO/ZnO:Zn heterostructure meets the requirement for the bilayer and, as a consequence, is a good candidate for stable memristive systems.

### 3.3. SEM Micrographs, EDS Analysis, and Substrate Temperature Model

**Figure 5:**
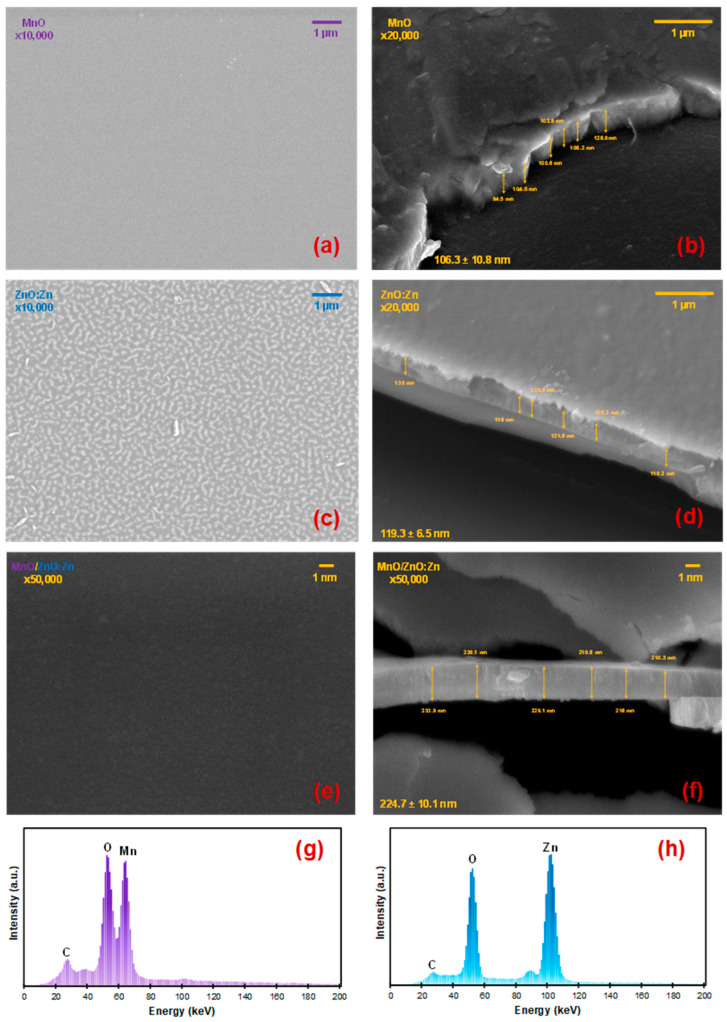
(**a**) SEM micrograph of surface of MnO (MnGl) at ×10,000, (**b**) cross-sectional image of MnO at ×20,000, (**c**) micrograph of surface of ZnO:Zn (ZnGl), (**d**) cross-sectional image of ZnO:Zn at ×20,000, (**e**) micrograph of surface of structure MnO/ZnO:Zn at ×50,000, (**f**) cross-sectional image of structure MnO/ZnO:Zn at ×50,000, (**g**) EDS analysis of MnGl, (**h**) EDS analysis of ZnGl.

For a morphological and thickness analysis of the samples, SEM micrographs were obtained at ×10,000 and 5 kV for the surface analysis and at ×20,000 and 10 kV for the cross-section images of both the MnO (MnGl) and ZnO:Zn (ZnGl) layers. Additionally, images were taken at ×50,000 and 10 kV for the MnO/ZnO:Zn structure. Figure 5 shows the surface of the samples in Figure 5a, Figure 5c, and Figure 5e respectively. The Mn deposition on glass is uniform and almost no defects are observed. The MnO film exhibits a characteristic mirror-like finish on glass, and upon application of the top ZnO:Zn film, no further oxidation is detected in the original film, which functions as a passivation layer [49]. Figure 5c shows the ZnO:Zn on glass where a labyrinth pattern can be detected [15]. The layer is transparent as already known in ZnO films, only with a certain tinted blue hue for the ZnO:Zn.

In Figure 5e, the surface of the MnO/ZnO:Zn also shows a homogeneous and smooth surface where the ZnO:Zn pattern prevails as is the top layer of the structure. A homogeneous surface ensures consistent electrical properties across the film, and the smoothness minimizes defect sites and irregularities that could lead to unpredictable switching behaviour or degrade the device response over time.

For the film thickness by SEM, Figure 5b,d,f show MnO, ZnO:Zn, and the final heterostructure of MnO/ZnO:Zn, respectively. The average thickness is determined by six measurements of each layer, resulting in 106.3 ± 10.8 nm for MnO, 119.3 ± 6.5 nm for ZnO:Zn, and 224.7 ± 10.1 nm for the final MnO/ZnO:Zn structure. These results closely align with those obtained by the profilometer for each layer, thereby corroborating the information provided by both the profilometer and the sputtering thickness monitor. Figure 5g,h present the EDS analysis of MnO and ZnO:Zn, respectively, showing that the materials of each film are present as the main elements, with no other atoms found aside from C.

**Figure 6:**
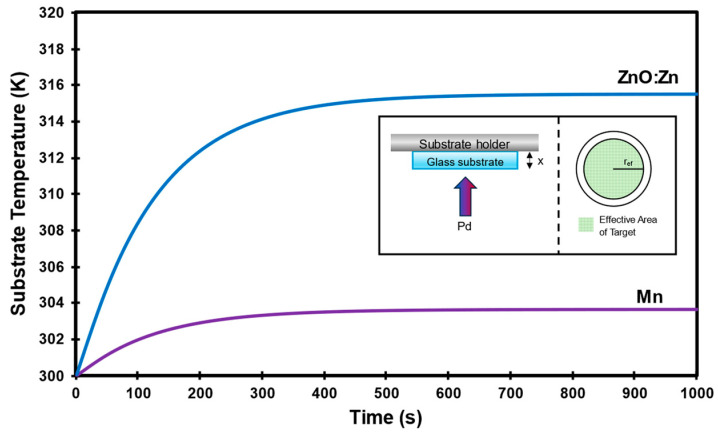
Solution of 1D heat equation for glass substrate for Mn and ZnO:Zn deposition. Stabilization time of deposition is around 500 s according to equation. Inset shows graphical model used in assumptions and effective area of a target.

By examining the morphological characteristics of the MnO/ZnO:Zn heterostructure and considering the room-temperature deposition process, we have established a substrate temperature model. Following the method of Khelfaoui and Aida [50], we employ a 1D heat equation model (Equation (4)) to calculate the surface temperature of the Mn and ZnO:Zn films during sputtering. This model allows us to estimate the films’ surface temperatures throughout the sputtering process, aiding in corroborating the low-thermal-impact assumption associated with room-temperature sputtering and deepening our comprehension of how temperature influences the deposition and, subsequently, the film’s final properties.

(4) $$\frac{\partial T}{\partial t}=\frac{k_{gl}}{C_{gl}\cdot \rho_{gl}}\frac{\partial^{2}T}{\partial x^{2}}$$

where $k_{gl}$ is the glass substrate thermal conductivity at 0.0014 $\frac{W}{cm\cdot K}$, $C_{gl}$ specific heat of the glass at 0.75 $\frac{J}{g\cdot K}$, and $\rho_{gl}$ is the density of glass at 2.5 $\frac{g}{{cm}^{3}}$. They indicated that the temperature mostly depends on the substrate material thermal conductivity and thickness [50]. For the 1 mm-thick glass substrate used, the following assumptions are considered for the model:

- There is no heating source for the substrate/substrate holder, so the initial temperature ($T_{0}$) is room temperature (fixed at 300 K).
- The energy flux from the targets and subsequently generated plasma is the heating source for the glass substrate and the temperatures are considered equal in the substrate for the growing process.
- The boundary conditions are:
  (5) $$T\left(x,0\right)=T_{0}$$
  (6) $$T\left(0,t\right)=T_{0}$$
  (7) $${\left.-\frac{dT\left(x,t\right)}{dx}\right|}_{x}=Pd$$
  where Pd is the power density of each target. While there are numerous interactions in the sputtering process (including sputtered atoms, Ar atoms, photons, etc.), we primarily consider the energy flux is mainly influenced by the power conditions of each target from the DC and RF sources. The effective area of the target is associated with the toroidal electromagnetic plasma generated during deposition. For our 2″ (approximately 5 cm) targets, a radius of 2 cm (effective radius—r_ef_) is utilized for the power density calculations provided in Table 5. For the co-sputtering process, the power densities were summed, although the 5 W contribution of Zn is minimal compared to the ZnO power. Figure 6 presents the results of the solved 1D heat equation as well as the graphical model illustrating the assumptions, where *x* represents the thickness of the substrate.

Based on the XRD results and with this model, it can be concluded that the temperatures of the substrate during the sputtering process do not exceed 320 K (47 °C), and the crystallinity of the samples indicates that, under the power conditions presented in this paper, the growth film does not exhibit significant defects or stress. Furthermore, SEM corroborates this observation, as the samples shown are homogeneous, and almost no rugosity is visible on the surface.

### 3.4. Frequency Adaptive Signal Processing

To investigate the adaptive behaviour of the MnO/ZnO:Zn, a bilayer structure was produced with the two-stage synthesis procedure described previously.

The interface operates under a dynamical polarization (time response), exhibiting both resistive and reactive (capacitive and inductive) impedance in the space-charge (SC) region. Therefore, to assess the technological capabilities of the MnO/ZnO:Zn heterostructure, the SC region of the heterojunction is analysed as a transfer function, defined as the ratio between the output and input signals.

Under the measurement technique shown in Figure 3, the dynamic behaviour of the MnO/ZnO:Zn structure can represent the correlation between the transfer function and the corresponding I–V characteristics to reveal internal states under specific operating conditions (in this case, changes in frequency).

The transient activation of states provides a qualitative understanding of the transport mechanisms involved in operating the MnO/ZnO:Zn heterojunction. The input consists of a sinusoidal signal of 4 V for each cycle, representing the condition for reproducible and repeatable states. At 4.8 V, the potential barrier is breached, resulting in a permanent loss of the rectifying behaviour.

**Figure 7:**
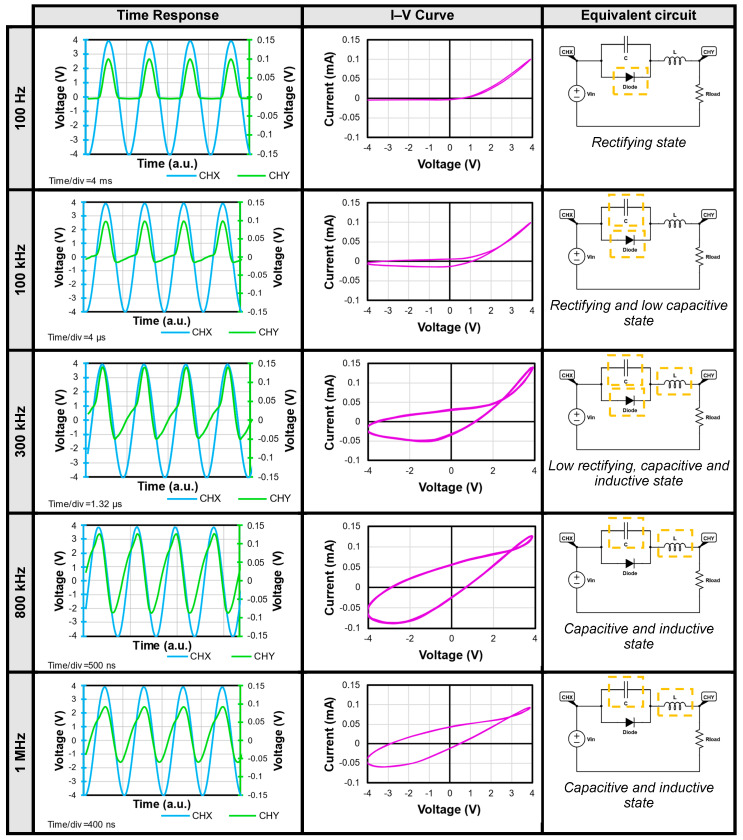
States of heterostructure where time response, I–V curve, and equivalent circuit are shown for each frequency (external stimulus). CHX and CHY are visible in time-response graph, each having a different scale given attenuation of output signal (CHY). I–V curves reveal evolution of space-charge region at each frequency. Analogy of the circuit is also presented, where simulation for a 1N4007 diode, L = 27 uH, C = 100 pF and Rload = 1 kΩ shows a similar output signal to ones of heterostructure at those frequencies. States are stable and can be achieved repeatedly under a signal of 4 V or less in the input.

The time-response signals, I–V curves, and equivalent circuit with the proposed contribution of each element are illustrated in Figure 7. The time is indicated as arbitrary units (a.u.) as the oscilloscope averages thousands of signals in each period. A time/div is displayed in each frequency to denote the equivalent period for each grid division on the time axis. It is important to note that the higher the frequency, the shorter the time, as there is an inverse relation.

An analysis of the performance at different test frequencies, ranging from 100 Hz to 1 MHz, follows. At lower frequencies (100 Hz to 10 kHz), a barrier forms at the interface of MnO/ZnO:Zn, causing the heterojunction to behave as a diode (half wave rectifier). However, at higher frequencies (100 kHz and 1 MHz), the rectifying behaviour changes due to the frequency increase, leading to a transformation of the signal. This change is reflected in the space charge region, which increases as the frequency rises. The hysteresis of the I–V curves is directly related to this alteration. A detailed explanation of the behaviour at each frequency is provided for better understanding of the frequency adaptive signal processing of the device.

At 100 Hz, a rectifying and resistive switching state is observed, with the threshold voltage of the device measured at 440 mV (sinusoidal input). This indicates the onset of diode response, which remains consistent across all frequencies. A filament conduction is presumed at the interface. The presence of filament conduction at the interface is presumed, as evidenced by the time response signal of forward bias rectification and the I–V curve. The rectification originates from the interface between the MnO and ZnO:Zn layers, where a certain number of electrons and holes have flowed, generating the electrical field of the space charge region. Consequently, this resistive switching state can be attributed to space-charge limited currents (SCLC), characterized by charge trapping, leading to a transition from a high-resistance state (HRS) to a low-resistance state (LRS) under forward-bias conditions. This transition corresponds to bipolar resistive switching. From 100 Hz to almost 100 kHz, the signal behaviour resembles that of a rectification-only state.

For 100 kHz, the capacitive rectifying state expands from −4 V to 4 V, indicating that the velocity of injected carriers is lower than the velocity of charge trapping. This extension of the state encompasses the reverse bias region (negative cycle), manifesting as a broadening in the I–V curve within the same voltage range. During this period, there is a transient accumulation of electron and hole carriers around the interface, accompanied by additional charge trapping in the space charge (SC) region. As a result of this transient condition, the SC region extends (with a corresponding reduction in the electric field), leading to a random distribution of carriers in the ZnO:Zn region beyond the interface. This distribution may contribute to the slow ionization of defects, thereby inducing a hysteresis phenomenon in the I–V curve.

In 300 kHz, the rectification effect becomes nearly imperceptible, and a crossing is observed in the hysteresis curve. This phenomenon, known as pinched hysteresis, is characteristic of ideal memristors, which exhibit a zero-crossing pinched hysteresis crossing at I = 0 and V = 0 (Table 1). However, in several reported structures, meminductive and memcapacitive effects can occur at interface [51,52]. In the case of the MnO/ZnO:Zn heterojunction, the pinched response is shifted and asymmetric, indicating imperfect memristors with a non-zero crossing I–V hysteresis [10,12,53]. This suggests that the MnO/ZnO:Zn heterojunction should be considered as an extended memristive device.

**Figure A1:**
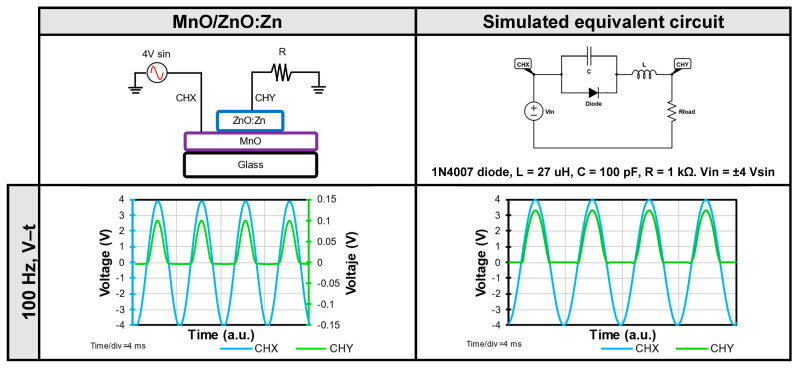

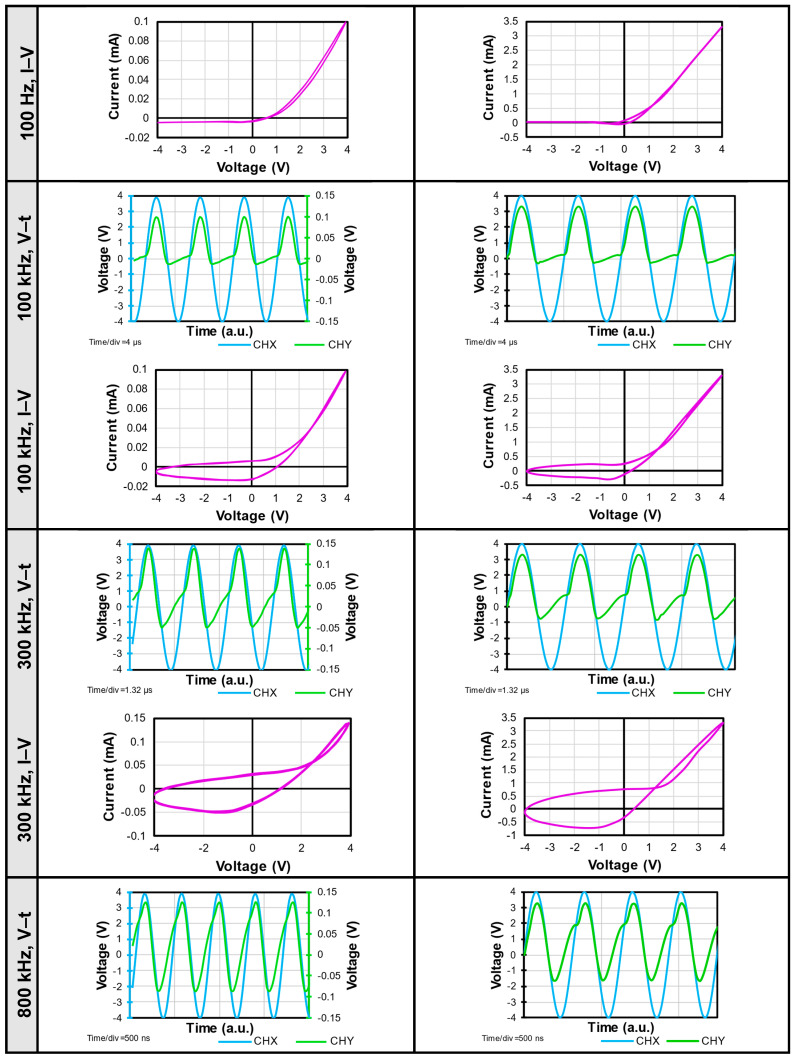

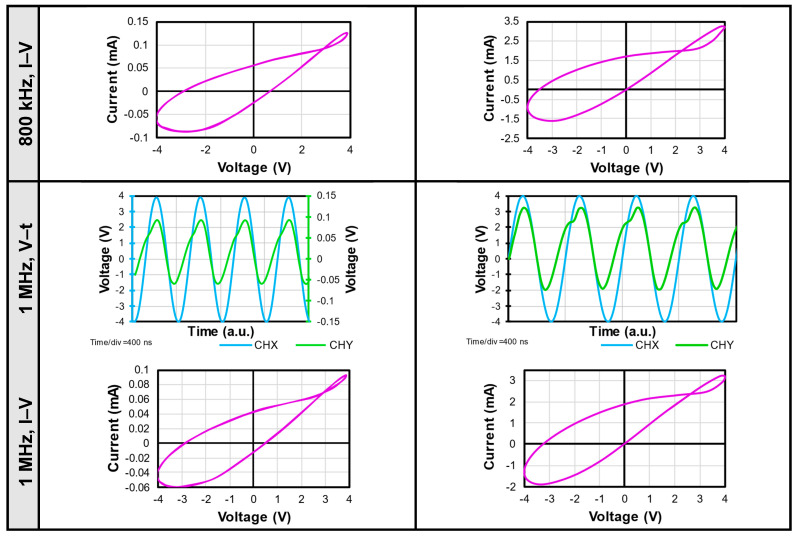
Comparison of V–t and I–V curves of simulated circuit in SPICE versus MnO/ZnO:Zn response.

At 800 kHz and 1 MHz, the time-response signal does not deliver a rectifying behaviour anymore. A greater capacitive response than the one at 300 kHz is present, as evidenced by the delay of the signal in the negative cycle of CHY relative to CHX. When the signal rises (negative to positive), the capacitive contributions are greater than the resistive ones. On the contrary, when the signal falls (positive to negative) the contributions are almost purely resistive. This capacitive response can be attributed to an expansion of the space charge (SC) region, where holes fill more rapidly due to the higher frequency of the state. This phenomenon is also reflected in the broader hysteresis of the I–V curve. The capacitive response has been studied separately in other work for a similar structure [54]. Appendix B, Figure A1 presents the comparison of the V–t and I–V curves of a simulated circuit compared to the MnO/ZnO:Zn heterostructure for further exploration of the equivalent circuit.

**Figure 8:**
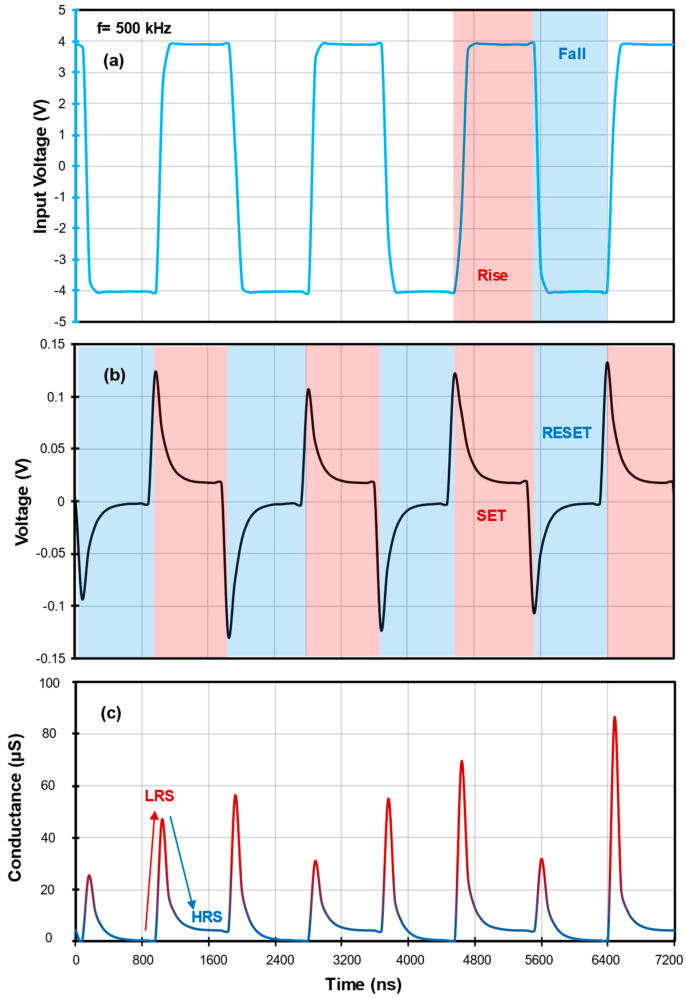
Applying a ±4V square signal to MnO/ZnO:Zn structure, we obtain (**a**) input signal with rise and fall signals indicated in red and blue, respectively; (**b**) response of structure as pulses, where the switching speed is measured in oscilloscope and indicated as 17 ± 0.82 ns for HRS to LRS and 439 ± 21.9 ns for LRS to HRS; and (**c**) conductance for applied pulses where switching ratio is calculated as 2.11 × 10^4^ ± 6.59 × 10^4^.

To analyse the transient switching characteristics of the memristor, a square signal of ±4 V is used as a pulsed signal at 500 kHz with the same configuration as before. Figure 8a illustrates the input signal, while the response of the structure is depicted in Figure 8b and the conductance for the applied pulses is showed in Figure 8c.

Figure 8a depicts the rise/SET and fall/RESET characteristics of the square signal to simulate pulses at 500 kHz. This frequency was chosen due to its features being representative of both 300 and 800 kHz. In Figure 8b, the response signal was used to measure the switching speeds in the oscilloscope using cursors. The determined values were 17 ± 0.82 ns from HRS to LRS and 439 ± 21.9 ns from LRS to HRS. These speed disparities not only emphasize the heterostructure’s quick adaptability to changing electrical conditions but also point towards its potential applications where fast switching mechanisms are needed. Using Ohm’s law (V=IR), the resistance values (V as the input voltage and I as the relation of the response voltage and the R_load_ of 1 kΩ), were calculated to obtain the conductance of each pulse (conductance is the inverse of resistance and measured in Siemens, $S=\Omega^{-1}$). Lastly, the switching ratio value was estimated as 2.11 × 10^4^ ± 6.59 × 10^4^. This value, reflective of the device’s ability to differentiate between resistance states, serves as a fundamental parameter for optimizing the material’s nanocomposition and configuration to enhance its memristive efficiency. Appendix C presents the power consumption of the device at different frequencies for additional efficiency parameters.

Finally, the heterojunction does not degrade with the changes in frequency and the states remain repeatable over the spectrum of 100 Hz–1 MHz (meaning that, for example, a jump from 1 MHz to 100 Hz, does deliver the same signal seen in each state). Both the external stimulus of the frequency and the stable, repeatable signals of each state, allows us to determine that the MnO/ZnO:Zn heterostructure can be deemed as a first approach to future sustainable frequency-adaptive memristive systems.

## 4. Conclusions

A simple MnO/ZnO:Zn heterostructure of approximately 200 nm was synthesized by sputtering and was found to have characteristics of extended memristive devices through frequency analysis. Unlike conventional multilayer memristive systems, this heterostructure capitalizes on a single interface of two materials, streamlining the complexity typically associated with such devices. XRD data show a MnO phase with high texture in (111) whereas the ZnO:Zn film is preferential towards (002) and the doped Zn atoms are introduced as interstitial Zn (Zn_i_) which enhances the conductive behaviour of the film in comparison to pure ZnO. SEM showcases a uniform and smooth surface across both materials, a critical attribute for ensuring consistent device performance.

Electrical characterisation, using an oscilloscope and function generator in the range of 100 Hz to 1 MHz, revealed a non-zero crossing I–V hysteresis, as well as capacitive effects associated to the interface, thus demonstrating the frequency adaptability of the system. The responses are stable, repeatable, and reproducible as long as the bias voltage does not exceed the ±4 V limit. The simple structure allows the analysis of an equivalent circuit model with the purpose of understanding the internal switching adaptive mechanisms through frequency. The switching speed from HRS to LRS of 17 ± 0.82 ns, highlight the heterostructure’s potential in fast-switching applications. Furthermore, the switching ratio of 2.11 × 10^4^ ± 6.59 × 10^4^, opens possibilities for optimising the material’s composition and structure to enhance this parameter, such as the improved conductance of the ZnO:Zn which can be exploited for future devices in which conductive, transparent, and easy to deposit ZnO films could be of use for future memristive applications.

As the field of frequency-adaptive devices emerges, the unique interaction between MnO and ZnO:Zn oxides has great potential for the development of new applications and technologies to transition from traditional to sustainable electronics. This study illustrates the potential of leveraging simple heterostructures as an option to create new and innovative design schematics with reduced components and complexity, paving the way for the next generation memristive devices.

## Figures and Tables

**Table 2:** Parameters for thin-film deposition.

Sample	Sputtering Target	Power Source	Ar Atmosphere	Working Pressure
MnGl	Mn, 99.9% purity	DC, 30 W	5 SCCM	3.5 mTorr
ZnGl	ZnO, 99.99% purity	RF, 125 W	10 SCCM	5 mTorr
	Zn, 99.999% purity	DC, 5 W	10 SCCM	5 mTorr

**Table 3:** Crystallite size (*D*), strain (*ε*), 2θ (measured θ_m_ and reference, θ_r_) and 2θ displacement (Δθ).

Plane	Phase	2θ_m_ (°)	2θ_r_ (°)	Δθ	*D* (nm)	*ε* (%)
(111)	MnO	34.448	34.446	0.002	10.3	−80 × 10^−6^
(002)	ZnO	34.133	34.422	−0.289	16.14	−0.77

**Table 4:** Parameters of interplanar distance (*d*) for MnO (bottom) and ZnO:Zn (top) films.

Plane	Layer	*d* (Å)	Δ*d*/*d*_MnO_ (%)
(111)	MnO	2.601	-
(002)	ZnO:Zn	2.623	0.846

**Table 5:** Power density of each target used in MnO/ZnO:Zn heterostructure.

Target	Power (W)	Power Density, Pd (W/cm^2^)
Mn	30	2.4
ZnO	125	9.94
Zn	5	0.4

## Data Availability

Data are contained within the article.

## Appendix A. Hall Effect Measurement

An electrical characterisation of each layer was made by Hall effect employing the Van der Pauw method using a magnetic field of 0.55 Tesla.

The information from the Hall measurements allows to know the carrier density, mobility, and resistivity of each film. In the ZnO:Zn sample, the Zn incorporation decreases the resistivity and can be measured in comparison to a ZnO film at the same conditions of synthesis [46]. Table A1 indicates the values obtained.

**Table A1:** Parameters obtained from Hall measurements for MnGl and ZnGl samples.

Sample	Type	Carrier Density (cm^−3^)	Mobility (cm^2^/V-s)	Resistivity (Ω-cm)
MnGl	p	3.40 × 10^21^	0.00762	0.2385
ZnGl	n	3.00 × 10^16^	9.2182	24.6547

The difference in semiconductor types for each film can be seen from the results of Table A1. The structure can take advantage of the p-n heterojunction type for a reconfigurable device. For the MnO layer, the main carriers will be the holes (h+) acting as acceptors and for the ZnO:Zn layer, the carriers will be the electrons (e−) acting as donors.

## Appendix B. Simulation of Equivalent Circuit

The comparison between the measured MnO/ZnO:Zn structure and the simulated equivalent circuit with the simulation program with integrated circuit emphasis (SPICE) is shown in Figure A1. The V–t and I–V curves are similar, and the main change is the intensity of the signals as the simulated signals show a minor attenuation in voltage and current. This circuit analogy allows us to observe what the behaviour of the interface between MnO and ZnO:Zn may be like with passive and active elements.

## Appendix C

The power consumption can be calculated as dynamic power consumption (Equation (A1)) given the frequency change at different states:

(A1) $$P=CV^{2}f$$

As we have the capacitor (*C*) value from the equivalent circuit as 100 pF, the frequency is our variable and Table A2 shows the power consumption at each frequency.

**Table A2:** Dynamic power consumption of MnO/ZnO:Zn structure at different frequencies.

Frequency	Dynamic Power Consumption
100 Hz	160 nW
100 kHz	160 µW
300 kHz	480 µW
500 kHz	800 µW
800 kHz	1.28 mW
1 MHz	1.6 mW

As the frequency rises, so does the power consumption. This is a direct relation of Equation (A1) and can be explained as: the more signal that is supplied to a device at higher frequencies, the input signal is faster and repeated more times than at a lesser frequency, increasing the consumption of energy.
